# Vibration-based condition monitoring, performance and emission evaluation of a diesel engine fueled with Karanja biodiesel

**DOI:** 10.1038/s41598-026-56375-y

**Published:** 2026-06-17

**Authors:** Vijay Kumar, Saurabh Dhiman, Amit Sanyal, K. Vijetha, Akhilesh Kumar Choudhary, Pawan Kumar Singotia, Abhishek Sharma, Alok Kumar Ansu

**Affiliations:** 1 Department of Mechanical Engineering, National Institute of Technology Hamirpur, Hamirpur, Himachal Pradesh 177005 India; 2 Department of Mechanical Engineering, Aditya University, Surampalem, Andhra Pradesh 533437 India; 3 Department of Mechanical Engineering, Raghu Engineering College, Vishakhapatnam, Andhra Pradesh 531162 India; 4 Department of Mechanical Engineering, Government Engineering College Palamu (Department of Higher and Technical Education, Government of Jharkhand), Medininagar, Jharkhand 822118 India; 5 Department of Mechanical Engineering, Manipal University Jaipur, Jaipur, Rajasthan 303007 India

**Keywords:** Accelerometer, Condition monitoring, Emission reduction, Engine vibration, Sustainable alternative fuels, Energy science and technology, Engineering

## Abstract

This study aims to experimentally investigate the performance, combustion, emissions, and vibration characteristics of a single-cylinder, four-stroke, water-cooled variable compression ratio (VCR) diesel engine fueled with diesel and Karanja biodiesel blends (B20 and B30). Experiments were conducted under varying engine load, compression ratio (CR), and hot exhaust gas recirculation (EGR-HOT) conditions. Engine vibration was evaluated using root mean square acceleration (RMS Accel), and a novel integrated approach was adopted to correlate vibration behavior with performance and emission characteristics. The results show that vibration increases with engine load but decreases with higher compression ratios, while under EGR conditions it initially decreases and then rises at higher rates. The results show that vibration increases with engine load but decreases with higher compression ratios, while under EGR-HOT conditions it initially decreases and then rises at higher rates. Compared to diesel, RMS acceleration decreased by 4.3 and 8.49% under load variation, 3.01 and 7.18% under CR variation, and 2.66 and 6.75% under EGR-HOT conditions for B20 and B30, respectively. Emission analysis revealed reductions of 13.3% in hydrocarbon (HC), 6.58% in carbon monoxide (CO), and 17.98% in smoke opacity for B30, although nitric oxide (Nox) increased by 15.8% as compared to diesel. Combustion analysis indicated a 3.43% increase in maximum cylinder pressure (CPMax) and a 14.94% decrease in net heat release (NHR). In terms of performance, brake thermal efficiency (BTHE) decreased by 10.74%, while brake-specific fuel consumption (BSFC) increased by 18.52%. Overall, the study demonstrates that biodiesel blends improve emission and vibration characteristics but slightly compromise performance, while the integrated analysis provides deeper insight into combustion behavior and engine condition.

## Introduction

Diesel engines are preferred in transport and stationary applications due to their robust construction, high load tolerance, efficient fuel consumption, and low maintenance demand^1,2^. They offer high brake thermal efficiency (BTHE) and strong torque characteristics; however, they also suffer from challenges such as elevated nitric oxide (NOx) and particulate emissions, along with significant engine vibrations. These vibrations adversely affect engine durability, noise levels, and operator comfort. With increasing concerns over environmental pollution and engine health, the investigation of alternative fuels for diesel engines has gained importance. Conventional diesel combustion contributes to harmful emissions and intensified vibration levels. Therefore, developing eco-friendly fuel alternatives that can simultaneously reduce exhaust emissions and vibration is essential. Within this framework, biodiesel obtained from a wide range of feedstocks has arisen as a promising alternative for conventional diesel^3–6^. Condition monitoring refers to the sophisticated machine monitoring that permits predictive maintenance and, as a result, the capability to identify an early flaw before it results in a noticeable problem. Early problem detection provides a number of advantages, including the ability to recognize a weakness before the primary flaw drives a secondary one and a major flaw occurs^7,8^. Therefore, condition monitoring of engine using accelerometer sensor for vibration data acquisition for early fault detection and to know the performance and health of an internal combustion (IC) engine by analyzing engine vibrations caused by combustion process and other means is becomes of importance^9^. Vibration Signature-Based Condition Monitoring involves assessing machine health by capturing vibrations from various events during operation, like combustion or fluid flow, using transducers. Machines, comprising prime movers, shafts, and rotating parts, generate these vibrations. The signals obtained offer valuable insights into machine health, enabling proactive maintenance and minimizing downtime^10^. Choudhary et al.^11^ provides good insight into this and studied that the engine structure has three main channels for the transmission of vibrations. Vibrations initiated in cylinder head propagate along engine block & dissipate into the environment. The cylinder block that transmits the vibrations is excited by gas explosions directly in the second route. Vibrations from the engine’s moving parts, such as connecting road and piston, are transferred to the engine block via the crankshaft in the third way, which is referred to as mechanically tempted vibration. The entirety of engine’s vibration & noise levels is instigated by combustion, which is released into the atmosphere through the engine’s external surfaces. These vibrations, which are often recorded from the cylinder head or engine block, include information about the combustion event^12^.

Ramachandran et al.^13^ offers a thorough analysis of the various sources and mechanisms of IC Engine vibrations, giving importance to effective vibration control and mounting systems. This paper talks about the consequences of unchecked vibrations, including accelerated wear and tear of engine components, increased maintenance costs, and reduces engine lifespan. Klinchaeam et al.^14^ utilizing vibration signals for condition monitoring of a 4-stroke gasoline engine. The present research employs crank-angle-resolved and time-domain vibration analysis techniques to observe the condition of a 4-stroke gasoline engine. The engine’s acquired vibration signals can be used to investigate abnormalities in the engine cycle and engine processes, such as the operation of the exhaust and valves, the ignition and combustion processes etc. Cheng et al.^15^ used vibration acceleration measurements from a diesel engine to determine combustion timing. They developed a finite element model to investigate correlation among the 2nd derivative of in-cylinder pressure & vibration acceleration at cylinder head. Winton and Dowling^16^ assessed engine vibration behavior by installing the engine block on multi-axis force transducers in a dynamometer test cell. Three modal analysis tests were conducted by exciting the left rear mount in three orthogonal impact directions and simultaneous recording of nine outputs as part of their technological approach. The engine block’s rigid body modal properties and force signatures from the engine mounts corresponding to six rigid body vibration modes that were detected were revealed by their findings, which used standard modal analysis techniques. Chiatti et al.^17^ analyzed engine acoustics & vibrations for small diesel engine running on biodiesel mixtures from recycled cooking oil and gasoline with ultra-low sulfur diesel. The results indicated that, RMS vibration amplitude enhanced with engine rate. Among tested fuels, B10 showed the lowest RMS vibration levels, whereas B40 produced the uppermost root mean square acceleration (RMS Accel) under diverse engine loads. Ong et al.^18^ investigated diesel engine vibrations using Calophyllum inophyllum biodiesel blends (B5–B30). At 2400 rev/sec, The B20 fuel showed a reduction in vibration, with a mean entire RMS Accel of 27 m/s², relative to neat diesel. Murugan and Ganesan^19^ reported that methanol-added WFO biodiesel ternary blends improved combustion performance, with B30M20D50 showing higher peak cylinder pressure than diesel, while vibration increased with higher heat release rates. However, higher methanol content reduced noise and vibration levels, making B20M30D50 more favourable from an NVH perspective despite slightly lower combustion performance. Kuruba and Lawrence^20^ evaluated combustion, performance, emissions, and vibration characteristics of a diesel engine using JUB20 biodiesel with hydrogen addition and modified piston geometries (SSVP and SSCVP), observing improved turbulence and combustion efficiency. The JUB20 + HYD + SSCVP configuration showed the highest BTHE (9.82%) and lowest brake specific fuel consumption (BSFC) (21.62%), along with reduced carbon monoxide (CO), carbon dioxide (CO₂), and hydrocarbon (HC) emissions, though with a slight increase in NOx and notable changes in vibration response. Abdo et al.^21^ investigated engine roughness, combustion noise, and vibration characteristics of a direct injection (DI) diesel engine using Jojoba biodiesel–diesel blends under varying load and speed conditions. Results showed that pure Jojoba biodiesel produced higher pressure rise rates, combustion noise, and RMS vibration compared to diesel, indicating increased engine roughness with higher biodiesel concentration. Zikri et al.^22^ reviewed the impact of biodiesel and its blends on diesel engine noise, vibration, and harshness (NVH) characteristics, highlighting how fuel properties such as cetane number, viscosity, and oxygen content influence combustion and acoustic behavior. The study concluded that lower blends generally reduce noise and vibration, while higher blends introduce trade-offs, and emphasized the need for standardized NVH methodologies and advanced analysis techniques.

Various researchers have examined diesel engine performance parameters, including BSFC and BTHE. Chen et al.^23^ found that exhaust gas recirculation (EGR) drops NOx emissions by recirculating exhaust gases through the intake manifold, decreasing combustion pressure and temperature. With EGR rates of up to 20% are achievable, although greater levels can degrade performance and raise other pollutants. A 15% EGR rate is typically utilised to balance oxides of nitrogen (NOx) reduction and engine efficiency. Pradeep and Sharma^24^ effectively utilized hot exhaust gas recirculation (EGR-HOT) to reduce NOx emissions in a diesel engine fueled with Jatropha biodiesel. Among the tested EGR-HOT percentage (5–25%), 15% was identified as the optimal rate, offering substantial NO reduction. Along with lower smoke, CO, and HC emissions, while maintaining satisfactory BTHE. Baweja et al.^25^ conveyed that maximum BTHE of a mustard biodiesel blend (B20) at full load was comparable to diesel, with only a 4.96% reduction observed for diesel operation. Under the same conditions, the BSFC of B20 was nearly identical to diesel. Verma et al.^26^ found that although biodiesel mixtures generally exhibit more BSFC than D100, the addition of eucalyptus biodiesel improved BTHE. Blends containing up to 10% biodiesel showed BTHE values comparable to diesel. Under 100% load conditions, BTHE values of 26.33%, 26.22%, 26.05%, and 25.54% were recorded for B10, B30, B50, and B100, respectively. In contrast, BSFC increased marginally from 268 g/kWh for diesel to 276.7 g/kWh for B10. Srivastava et al.^27^ carried study on diesel, roselle biodiesel blends (R20 and R30) and Karanja blends (K20 and K30). Under all loading scenarios, diesel fuel continuously showed the highest thermal efficiency. A full-load thermal efficiency of approximately 32.1% has been established for diesel fuel, while approximate thermal efficiencies of 32.8% and 32.5%, respectively, have been demonstrated for RB10 and RB20. It was found that the values of BSFC for RA10 and RA20 were, respectively, 5.45% and 6.5% more than those for diesel. Mathimani et al.^28^ observed maximum CO and HC emissions at full load for diesel & most biodiesel mixtures. However, B50 and B60 reduced HC emissions to 105 and 104 ppm, respectively, referred with 123 ppm for diesel, along CO emissions of 0.12% and 0.14% at 1500 rev/min. NOx emissions were alike to diesel fuel. Oh et al.^29^ reported HC emissions of ~ 27.55 ppm for petrol and diesel at 1900 rpm, while JCB50, CPB50, and CIB50 biodiesel blends exhibited higher emissions of 30.77, 30.88, and 31.91 ppm, respectively. On the other hand, HC generation was less than D100 when a 10% biodiesel blend was utilized. With respect to JCB10, CPB10, and CIB10, the corresponding values of HC formation at 1900 rpm were 18.68 ppm, 18.03 ppm, & 18.08 ppm. Compared to D100, NOx emissions increased. Aatia et al.^30^ found that HC production for B10 was 40% lesser than that of pure D100 due to high viscosity of B10 is offset by the oxygen concentration of CME. However, In comparison to B10, HC emissions are greater with higher biodiesel mixes, such as B20 and B30. The average decrease in CO emissions was between 14 and 18%. Akar et al.^31^ in their evaluation of WCO biodiesel blends discovered that, as compared to diesel, CO emissions for B10 & B20 decreased by 2.3% and 3.56%, respectively. The selection of only B20 and B30 blends may appear limited; however, it is based on a clear rationale. Extensive studies have already been conducted on lower blends such as B10 and B20, with the literature consistently identifying B20 as an optimal blend in terms of performance, emissions, and engine compatibility. Therefore, B20 was chosen as a baseline for this study. In addition, B30 was selected to further investigate the effects of a higher biodiesel proportion on engine performance, emission characteristics, and vibration behavior. This approach also enables a comparative assessment between a well-established optimal blend (B20) and a higher blend (B30) to better understand the influence of increased biodiesel content.

From the literature survey, it is clearly observed that performance, combustion, emissions, and vibration characteristics have mostly been studied separately for diesel engines using biodiesel fuels. Many researchers have reported performance and emission results, while some studies have focused only on vibration analysis for condition monitoring. However, a combined study of performance, combustion, emissions, and vibration behavior in a single framework is still missing. In particular, the combined effect of important operating parameters such as engine load, CR, and hot exhaust gas recirculation (EGR-HOT) on performance, combustion, exhaust emissions, and engine vibration has not been properly investigated. Because of this, the relationship between combustion process, emission formation, and vibration response is still not clearly understood. To fill this research gap, the present study carries out a combined and systematic investigation of a diesel engine fueled with Karanja biodiesel under different operating conditions. The main novelty of this work is that it studies performance, combustion, emissions, and vibration together, instead of treating them separately as done in most previous studies. In addition, this study connects vibration signals with performance and emission results, which helps in better understanding the combustion behavior and engine condition. This combined approach provides more complete information about how the engine works under different conditions. By analyzing the effects of load, CR, and EGR-HOT together, this study gives useful insights into combustion quality, emission formation, and vibration response. The connection between combustion behavior and vibration characteristics is established through parameters such as ignition delay, heat release rate, and in-cylinder pressure rise. Rapid and uncontrolled combustion leads to higher pressure gradients and increased vibrations, whereas smoother combustion results in reduced vibration levels. It also supports the development of vibration-based condition monitoring methods and helps in the effective use of Karanja biodiesel in diesel engines. The objectives of this study are as follows: (a) To study engine vibration behavior using RMS acceleration at different load, CR, and EGR-HOT conditions. (b) To evaluate engine performance and combustion under different operating conditions using Karanja biodiesel. (c) To analyze exhaust emissions under varying operating conditions using Karanja biodiesel.

## Material and methodology

### Preparation and properties of diesel and biodiesel blends

**Fig. 1:**
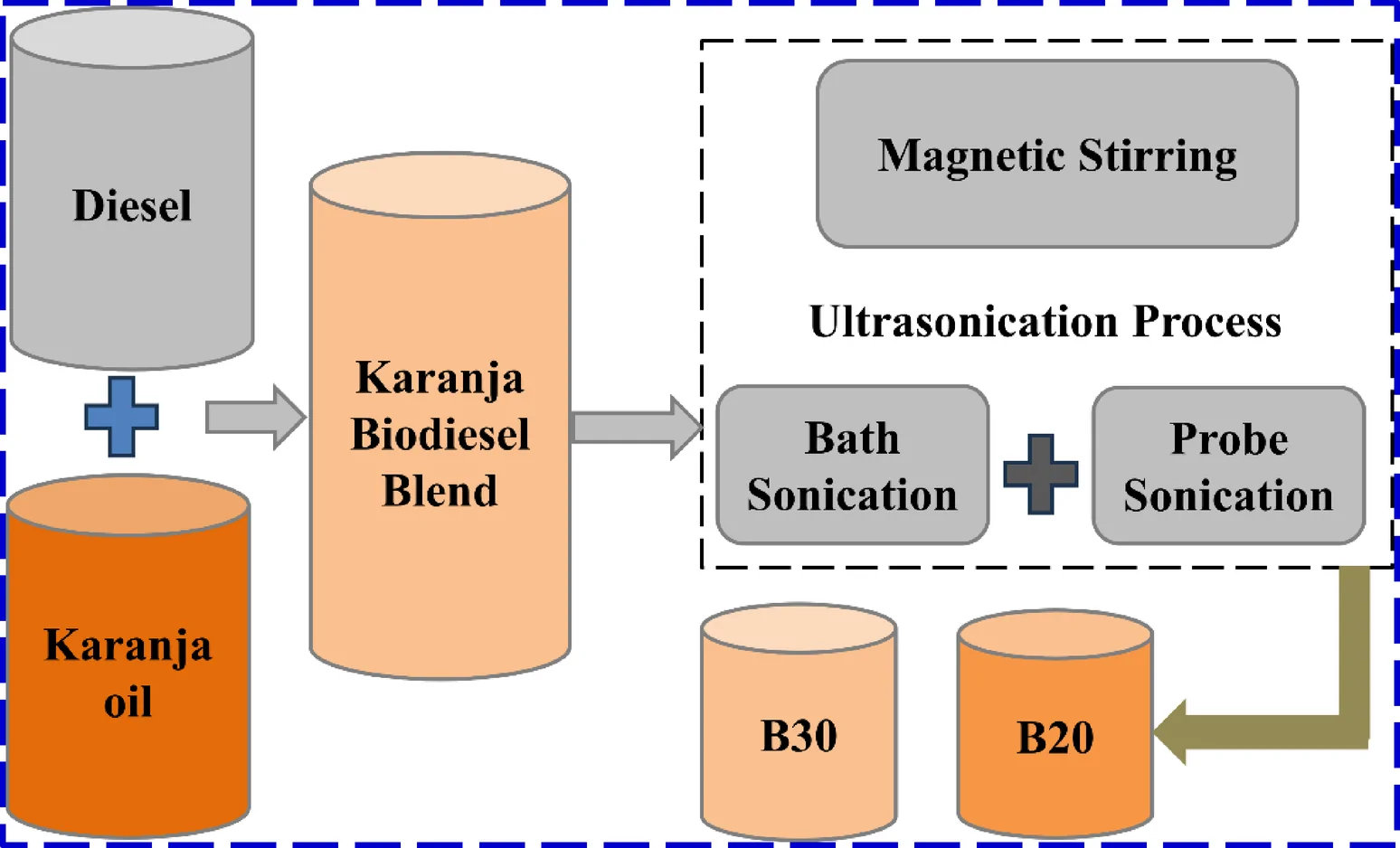
Biodiesel preparation.

Pure Karanja biodiesel (B100) was purchased from Dehradun at Paritosh Herbals; and diesel was bought from Reliance Petroleum pump, Hamirpur (HP). A quantity of 1.5 L for biodiesel and diesel each, was acquired & used to prepare the diesel-biodiesel blend, respectively. Fuels blends used for experiments: (a) Diesel (D100), (b) Karanja biodiesel blend (B20–80% diesel + 20% Karanja biodiesel) and (c) Karanja biodiesel blend (B30–70% diesel + 30% Karanja Biodiesel). To ascertain the physiochemical properties, like calorific value and density, ASTM standards were utilized & the values are shown below in Table 1. The preparation of the as shown in Fig. 1 biodiesel blends B20 and B30 was done by mixing Karanja biodiesel with a Vol% of 20 and 30% with 80% and 70% diesel respectively for the preparation of 1 L of B20 and B30 blends each. Further, to achieve a homogenous mixture, the mixture was heated to 50 degrees Celsius and bath sonification process was done for 1 h at ambient temperature. High-frequency sound waves are used in sonification to create cavitation bubbles that violently expand and contract, producing extreme mixing and microstreaming.

**Table 1:** Physio-chemical properties of different fuels.

Properties/fuel	ASTM	Diesel	B100	B20	B30
Calorific value (MJ/kg)	ASTM 4809	42	39.6	41.538	41.307
Density(kg/m^3^)	ASTM 4052	820	910	838	847
Viscosity (mm^2^/s)	ASTM 445	2.5	4.71	3.12	3.54
Cetane number	ASTM D613	48	52	48.64	49.16

### Vibration measurement, experimental Setup and experimental procedure

#### Vibration measurement methodology

**Fig. 2:**
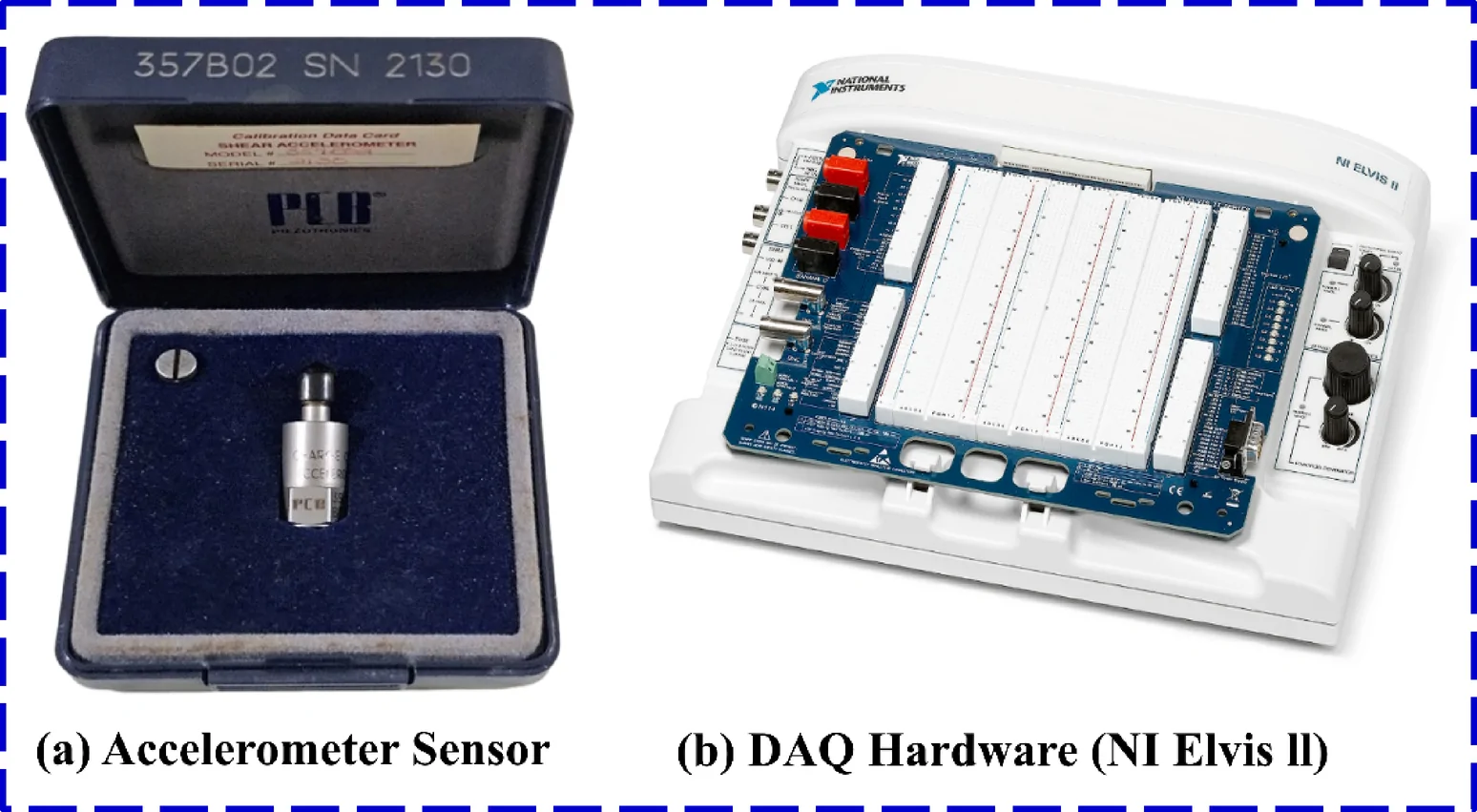
Accelerometer Sensor with NI ELVIS ll DAQ hardware.

The accelerometer was mounted on the cylinder head in the vertical direction, as this location is in close proximity to the combustion chamber and is highly sensitive to combustion-induced pressure fluctuations and structural responses. This placement enables effective capture of high-frequency vibration signatures associated with combustion events, ignition delay, knock characteristics, and fuel–air interaction. Moreover, mounting on the cylinder head minimizes signal attenuation and structural damping compared to locations such as the engine block or crankcase, thereby ensuring more representative and reliable vibration measurements. In digital signal processing, the Nyquist–Shannon sampling theorem states that a signal must be sampled at a rate at least twice its highest frequency component to accurately reconstruct the original signal and avoid aliasing. According to the accelerometer specifications, the maximum measurable frequency is 9000 Hz. Therefore, to satisfy the Nyquist criterion and prevent frequency distortion, the sampling rate was set at 16,000 Hz, which is approximately twice the sensor’s upper frequency limit. A sample size equal to half of the sampling rate was selected, corresponding to a data acquisition duration of 500 milliseconds. This approach ensures accurate capture of engine vibration signals while preserving signal integrity and avoiding overlap of frequency components. The calibration procedure of the accelerometer and data acquisition (DAQ) system has also been detailed. As per the calibration data card, the accelerometer sensitivity was provided in pC/g, which was converted into mV/g for compatibility with the LabVIEW program. This converted sensitivity was used as a constant (divide factor) to transform the measured voltage signals into acceleration units (g). This step was essential since the LabVIEW output is initially recorded in voltage form. The accelerometer was factory-calibrated, and its sensitivity was verified prior to experimentation. Additionally, the DAQ system was checked for signal accuracy and integrity. These steps ensure precise measurement of vibration signals and enhance the reliability and reproducibility of the experimental results. The accelerometer sensor with NI ELVIS ll DAQ hardware used in this study shown in Fig. 2. The specification of accelerometer sensor with NI ELVIS ll DAQ hardware has been displayed in Tables 2 and 3.

**Table 2:** Accelerometer specifications.

Model	357B02
Charge sensitivity	13.98 pC/g
High frequency limit	9000 Hz
Capacitance	729 pF
Manufacturer	PCB Piezotronics

**Table 3:** DAQ Hardware specifications.

Model	NI virtual instrumentation suite II (NI ELVIS II)
Maximum sampling rate	16-bit resolution at 100 kS/s
Analog input channels	16 differential channels
Frequency range	100 kHz
Connectivity and software	Integrates with LabVIEW software and includes USB connectivity for easy setup and data transfer.

#### Experimental setup and experimental procedure

**Fig. 3:**
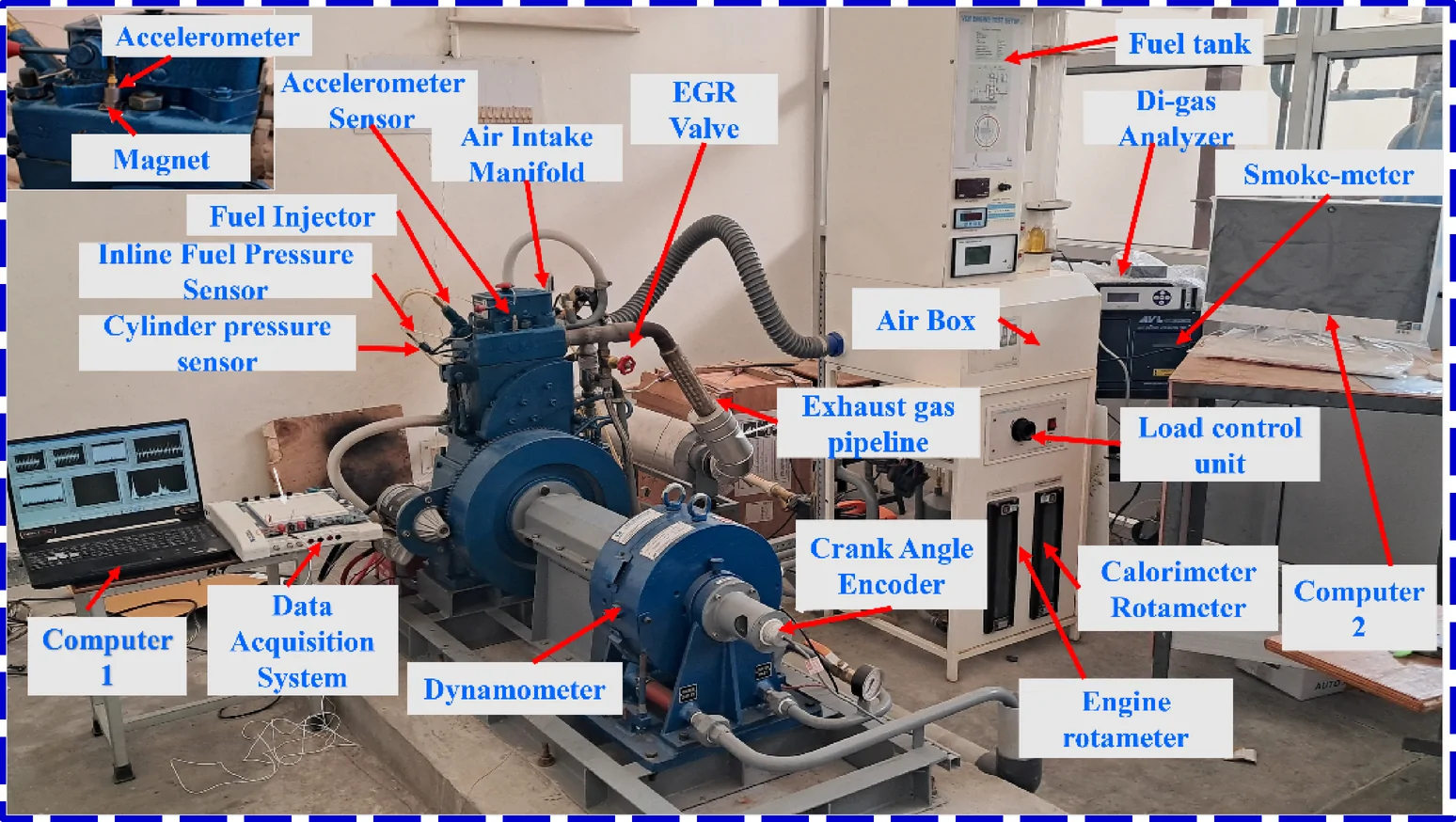
Experimental setup for engine vibration data acquisition.

A direct Injection, 4-stroke, single cylinder diesel engine has been used for experimental purpose and data has been collected. The experimental setup’s specifications are displayed in the table. An eddy current dynamometer was employed to apply torque & control engine load during experiments. The engine vibrations were calculated using an Accelerometer Sensor with NI ELVIS ll DAQ hardware as the interphase between sensor and computer. The magnetic mount method has been adopted to mount the sensor in the engine block for acquiring the vibrations signal during experiments it can be seen in the Fig. 3. A piezoelectric pressure transducer recorded the engine’s pressure readings during its 4-stroke cycle. The CO, NOx, and HC were among the untreated exhaust pollutants that were determined using the AVL DI gas analyzer. After completing experimental setup, tests were first conducted using diesel fuel under various conditions, including different loads, EGR-hot mode, and varying CR.

The exhaust gas from the exhaust outlet is directed into the EGR pipeline and subsequently transferred to the EGR chamber, where it mixes with fresh intake air before entering the intake manifold. The EGR quantity is controlled using an EGR valve, and its rate is determined indirectly through air flow measurements. When the EGR valve is fully closed, the total air flow to the intake manifold is measured as 29 kg/h using an air flow transmitter, corresponding to 0% EGR. For 7.5% EGR, the valve is gradually opened until the air flow decreases to 26.825 kg/h, as monitored through the data acquisition software. This reduction indicates that 2.175 kg/h of exhaust gas is being recirculated, corresponding to 7.5% EGR. Similarly, for 15% EGR, the valve is adjusted until the air flow reaches 24.65 kg/h, indicating that 4.35 kg/h of exhaust gas is recirculated, corresponding to 15% EGR. The air flow transmitter used in the study is calibrated. This methodology ensures precise control and repeatability of the EGR-HOT operating conditions.

After completing the experimental setup, baseline tests were conducted using diesel fuel under various operating conditions, including different loads, EGR-hot mode, and varying compression ratios. The engine was operated at a constant speed of 1500 rpm, with an injection pressure of 300 bar and injection timing set at 23° BTDC. For experimental purposes, the engine was started using diesel and maintained to establish a steady condition for 35 min at no load. As the engine achieved a steady state, diesel was switched to blended fuel, and the engine ran for 35 min to establish a steady state. Vibration, emission, combustion, and performance characteristics were evaluated under steady-state conditions across multiple test scenarios. These included varying load conditions (0, 25, 50, 75, and 100%), compression ratios (14, 15, 16, 17 and 18), and EGR-HOT levels (0%, 3.75%, 7.5%, 11.25%, and 15%). Measurements were taken for engine block vibrations primarily at the cylinder head as RMS Accel. The performance and combustion parameters like BTHE, BSFC, NHR and CPMax recorded. The emission parameters, including CO, NOx, HC and smoke opacity were also recorded. Each test condition was repeated five times to ensure accuracy, and the average of the recorded data was used for final analysis. The same experimental procedure was then repeated using the biodiesel blends B20 and B30 to complete the study. Table 4 shows the engine specification.

**Table 4:** Engine specifications.

Parameters	Engine specifications
Engine	Kirloskar TV 1
Details	1 Cylinder, 4-Stroke, Compression ignition (CI), Water-cooled
CR	Variable CR (12–18)
Bore/stroke	87.5 mm/110 mm
Rated power	3.5 kW at 1500 RPM
Swept volume	661.45 cc

### Uncertainty analysis

During data collection, uncertainties may arise from variations in engine operating conditions, limitations in measurement accuracy, human involvement, and testing procedures. To account for these factors, a systematic mathematical approach was employed to evaluate and quantify the overall experimental uncertainty.

$$\begin{gathered} {\text{Uncertainty in the overall experiment}} \hfill \\ ={\text{ }}\surd {\text{ }}\left[ \begin{gathered} {\left( {{\text{Systematic error of RMS Accel}}} \right)^{\mathrm{2}}}+{\text{ }}{\left( {{\text{Systematic error of Load}}} \right)^{\mathrm{2}}}+{\text{ }}{\left( {{\text{Systematic error of speed}}} \right)^{\mathrm{2}}} \hfill \\ +{\text{ }}{\left( {{\text{Systematic error of BTHE}}} \right)^{\mathrm{2}}}+{\left( {{\text{Systematic error of BSFC}}} \right)^{\mathrm{2}}}+{\text{ }}{\left( {{\text{Systematic error of CPMax}}} \right)^{\mathrm{2}}} \hfill \\ +{\text{ }}{\left( {{\text{Systematic error of NHR}}} \right)^{\mathrm{2}}}+{\left( {{\text{Systematic error of NOx}}} \right)^{\mathrm{2}}}+{\text{ }}{\left( {{\text{Systematic error of CO}}} \right)^{\mathrm{2}}} \hfill \\ +{\text{ }}{\left( {{\text{Systematic error of HC}}} \right)^{\mathrm{2}}}+{\text{ }}{\left( {{\text{Systematic error of Smoke opacity}}} \right)^{\mathrm{2}}} \hfill \\ \end{gathered} \right]. \hfill \\ ={\text{ }}\surd {\text{ }}\left[ {{{\left( {0.{\mathrm{1}}} \right)}^{\mathrm{2}}}+{\text{ }}{{\left( {0.{\mathrm{1}}} \right)}^{\mathrm{2}}}+{\text{ }}{{\left( {\mathrm{2}} \right)}^{\mathrm{2}}}+{\text{ }}{{\left( {0.{\mathrm{1}}} \right)}^{\mathrm{2}}}+{\text{ }}{{\left( {0.0{\mathrm{1}}} \right)}^{\mathrm{2}}}+{\text{ }}{{\left( {0.{\mathrm{1}}} \right)}^{\mathrm{2}}}+{\text{ }}{{\left( {0.{\mathrm{1}}} \right)}^{\mathrm{2}}}+{\text{ }}{{\left( {0.{\mathrm{6}}} \right)}^{\mathrm{2}}}+{{\left( {0.{\mathrm{2}}} \right)}^{\mathrm{2}}}+{{\left( {0.{\mathrm{3}}} \right)}^{\mathrm{2}}}+{{\left( {0.{\mathrm{1}}} \right)}^{\mathrm{2}}}} \right] \hfill \\ ={\text{ }} \pm {\mathrm{2}}.{\text{13 }}\% . \hfill \\ \end{gathered}$$

## Result and discussion

### Engine block vibration analysis for different fuel blends

#### Effect of operating parameters on RMS acceleration

**Fig. 4:**
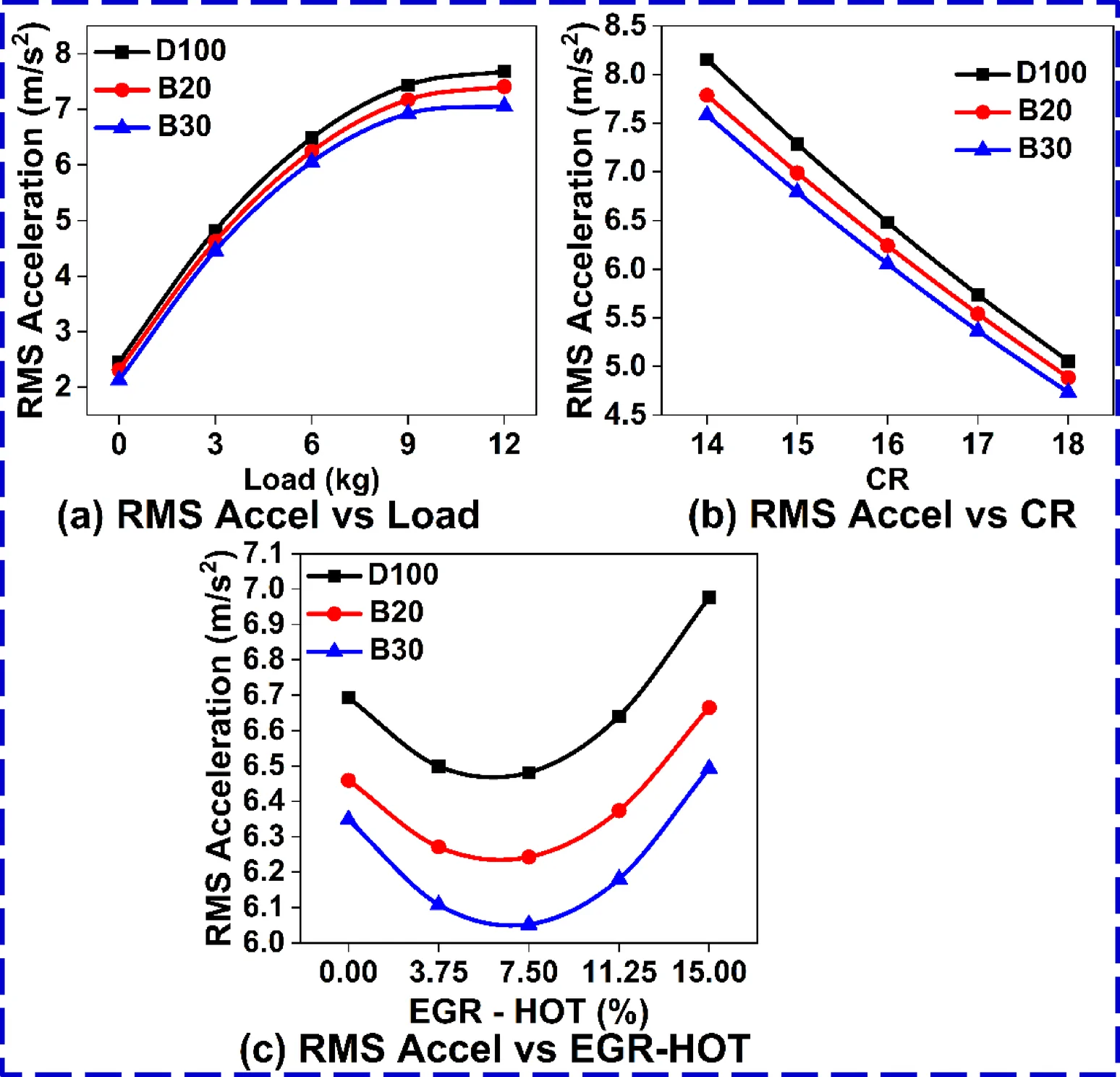
Significance of RMS Accel (**a**) RMS Accel vs. Load, (**b**) RMS Accel vs. CR, (**c**) RMS Accel vs. EGR-Hot.

Figure 4 (a) presents the RMS Accel response to engine load for diesel (D100), B20, and B30 at fixed CR of 16 and hot EGR of 7.5%. At 0% load, RMS Accel decreased progressively from diesel (2.456 m/s²) to B20 (2.315 m/s²) and B30 (2.131 m/s²), indicating reduced vibration intensity with increasing biodiesel content. At 100% load, the values were 7.681112 m/s², 7.407211 m/s², & 7.055956 m/s², respectively. On comparing the curves on the RMS Accel vs. Load plot for Diesel fuel with biodiesel blends (B20, B30) under varying loads from 0 kg to 12 kg, the graph shows a decreased in RMS Accel with biodiesel blends. The results show that RMS Accel increases with engine load for all fuel blends. They indicating a strong positive correlation between load & vibration intensity. Maximum vibrations are observed at a load of 12 kg, while the lowest values occur under no-load conditions. Higher loads increase fuel input to combustion chamber, leading to richer mixture & faster combustion rates, which promote knocking phenomena and consequently elevate engine vibrations. The average RMS Accel for B20 and B30 diminished by 4.3% & 8.49%, respectively, compared to diesel. This drop in vibrations is accredited to higher lubricity of biodiesel, which improves smoothness of engine operation. Lubricity influences vibration by reducing friction and wear between moving engine components, resulting in smoother mechanical operation and lower friction-induced vibrations. Cetane number plays a crucial role in combustion behavior by shortening the ignition delay and promoting a more controlled heat release rate, thereby minimizing abrupt pressure rise inside the cylinder. Together, these factors contribute to more stable combustion and reduced vibration levels. Furthermore, the higher cetane number of biodiesels supports more complete combustion, which helps in reducing combustion noise and vibrations. Additionally, the presence of inherent oxygen in biodiesel enhances combustion efficiency, leading to lower peak pressure fluctuations and smoother engine operation^32–34^. Thus, biodiesel blends not only provide environmental benefits but also significantly reduce engine vibrations compared to conventional diesel fuel, especially under varying load conditions.

Engine vibrations typically decrease with increase in CR. Figure 4 (b) illustrations variation of RMS Accel with fuel type for diesel (D100), B20, and B30 at a fixed load of 6 kg and hot EGR rate of 7.5%. At a CR of 14, the RMS Accel decreased from 8.156 m/s² for diesel to 7.786 m/s² for B20 and 7.583 m/s² for B30, indicating reduced vibration levels with increasing biodiesel content. At CR 18, values were 5.055191 m/s², 4.885011 m/s², & 4.733116 m/s², respectively, when comparing diesel fuel with biodiesel blends (B20, B30) the curves show a decreased in RMS Accel with biodiesel blends. The results indicate that engine vibration decreases with growing CR for all fuel blends, demonstrating clear correlation between CR & vibration intensity. Higher vibration levels are observed at CR of 14, while progressively lower values occur at higher CR. At lower CR, reduced in-cylinder temperature and pressure lead to less efficient combustion and increased ignition delay, which can promote knocking tendencies and result in elevated vibration levels. The average RMS Accel for B20 and B30 diminished by 3.01 & 7.18%, respectively, compared to diesel. Figure 4 (b) demonstrates that engine vibration decreases with growing CR for all fuel blends, indicating strong correlation between CR and vibration intensity. Higher CR increase in-cylinder pressure & temperature, which shorten ignition delay & suppress knocking tendencies, thereby reducing vibration levels^35^.

Exhaust gas recirculation (EGR) impacts engine vibrations in a two-phase manner. Initially, as the EGR percentage increases, engine vibrations decrease up to a mid-range EGR value. However, with further increases in the EGR percentage, engine vibrations begin to rise, peaking at the maximum EGR-Hot value. Figure 4 (c) illustrates variation of RMS Accel along hot EGR rate for D100, B20, & B30 at constant load of 6 kg and CR 16. At 0% hot EGR, RMS Accel values were 6.693 m/s² for diesel, 6.460 m/s² for B20, and 6.349 m/s² for B30. At the mid-range hot EGR level of 7.5%, RMS Accel decreased to 6.482 m/s², 6.242 m/s², and 6.052 m/s², respectively, indicating reduced engine vibrations. However, at higher hot EGR (15%), RMS Accel increased for all fuel blends, reaching 6.976 m/s² for diesel, 6.665 m/s² for B20, and 6.493 m/s² for B30. On comparing Diesel fuel with biodiesel blends (B20, B30) the curves show a decreased in RMS Accel with biodiesel blends. The average RMS Accel for B20 and B30 decreased by 2.66% and 6.75%, respectively, compared to diesel. Figure 4 (c) shows that engine vibration decreases with increasing hot EGR rate up to 7.5%, beyond which RMS Accel increases for all fuel blends. The initial reduction in vibration is attributed to improved combustion resulting from elevated intake charge temperature with hot EGR, along with a potential reduction in pumping losses due to slightly higher EGR pressure. However, hot EGR rates above 7.5% cause excessive charge dilution, leading to combustion deterioration and a consequent rise in RMS Accel^24^.

#### Engine block vibration analysis in time and frequency domains

**Fig. 5:**
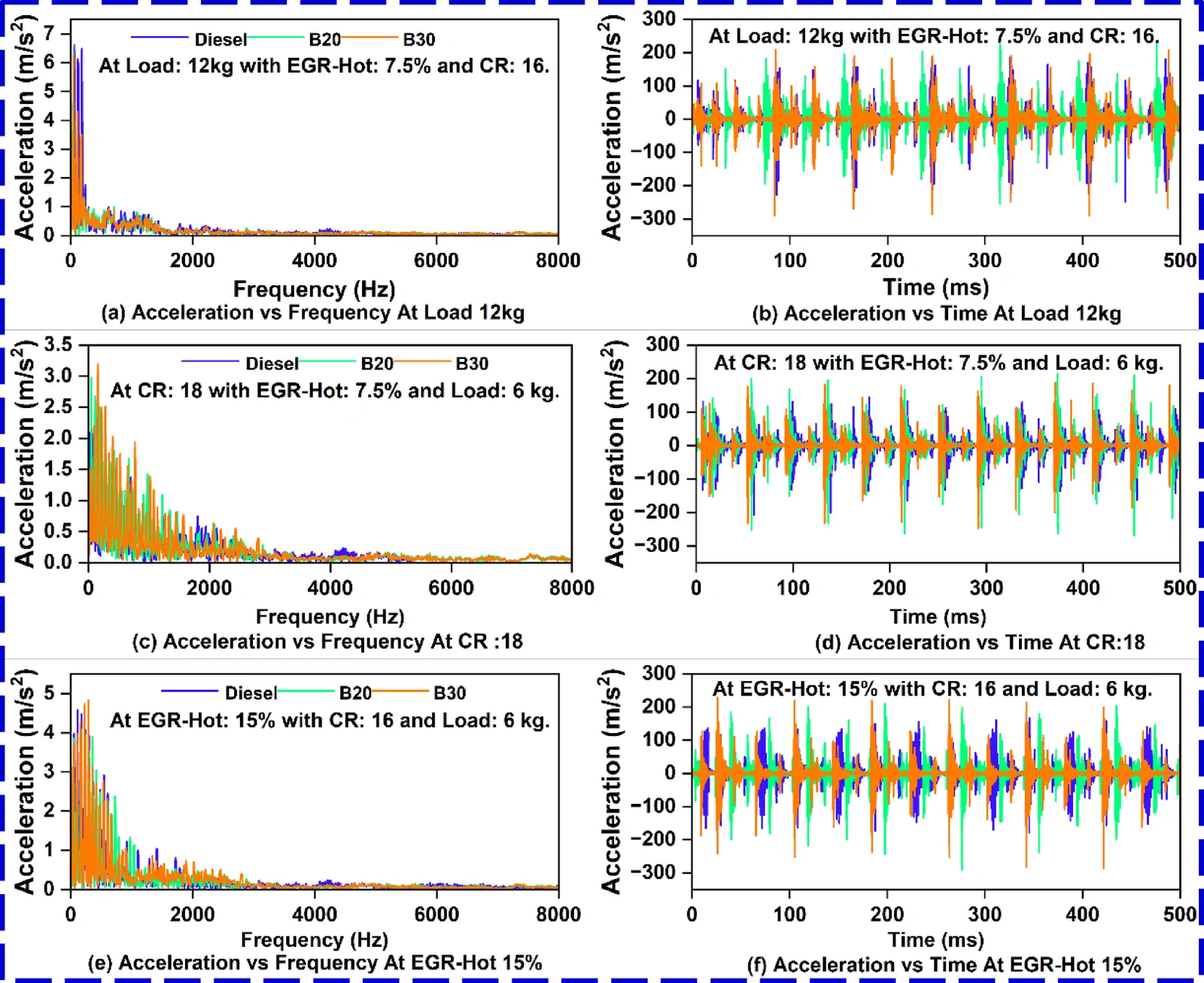
Effect of time and frequency on acceleration for different fuel blends (**a**) Acceleration Vs Frequency at load 12 kg, (**b**) Acceleration vs. Time at load 12 kg, (**c**) Acceleration vs. Frequency at CR 18, (**b**) Acceleration vs. Time at CR 18, (**e**) Acceleration Vs Frequency at EGR-Hot 15%, (**f**) Acceleration vs. Time at EGR-Hot 15%.

Figure 5 (a) illustrates the vibration spectra in terms of acceleration versus frequency at full load (12 kg) for different fuel blends, with the CR fixed at 16 and hot EGR at 7.5%.

Figure 5 (b) presents the acceleration–time vibration response at full load (12 kg) for different fuel blends, with the CR fixed at 16 and hot EGR maintained at 7.5%. Figure 5 (c) presents the acceleration–frequency vibration spectra at a CR of 18 for different fuel blends, with load fixed at 6 kg and hot EGR maintained at 7.5%. Figure 5 (d) shows the corresponding acceleration–time vibration responses under the same operating conditions. Figure 5 (e) presents the acceleration - frequency vibration spectra at a hot EGR rate of 15% for different fuel blends, with the load fixed at 6 kg and the CR maintained at 16. Figure 5 (f) illustrates vibration patterns on an acceleration - time spectra at 15% EGR-Hot with input factors Load 6 kg, CR 16 for different fuel blends. for various fuel blends. The acceleration–time histories represent the raw vibration signals for each fuel blend. Although similar fluctuation patterns are observed across all fuels, diesel exhibits marginally higher peak-to-peak amplitudes than the biodiesel blends. Together, the time- and frequency-domain analyses provide insight into the impact of fuel composition on engine vibration features. These spectra were obtained using Fast Fourier Transform (FFT) analysis to recognize and compare frequency components of the engine vibration signals. The results indicate that acceleration amplitude declines with growing frequency for all fuel blends. Diesel displays uppermost vibration peaks in the low-frequency range, whereas the biodiesel blends show comparatively lower peak amplitudes^33,36^.

### Impact of operating parameters on performance and combustion characteristics

#### Impact of operating parameters on BSFC

**Fig. 6:**
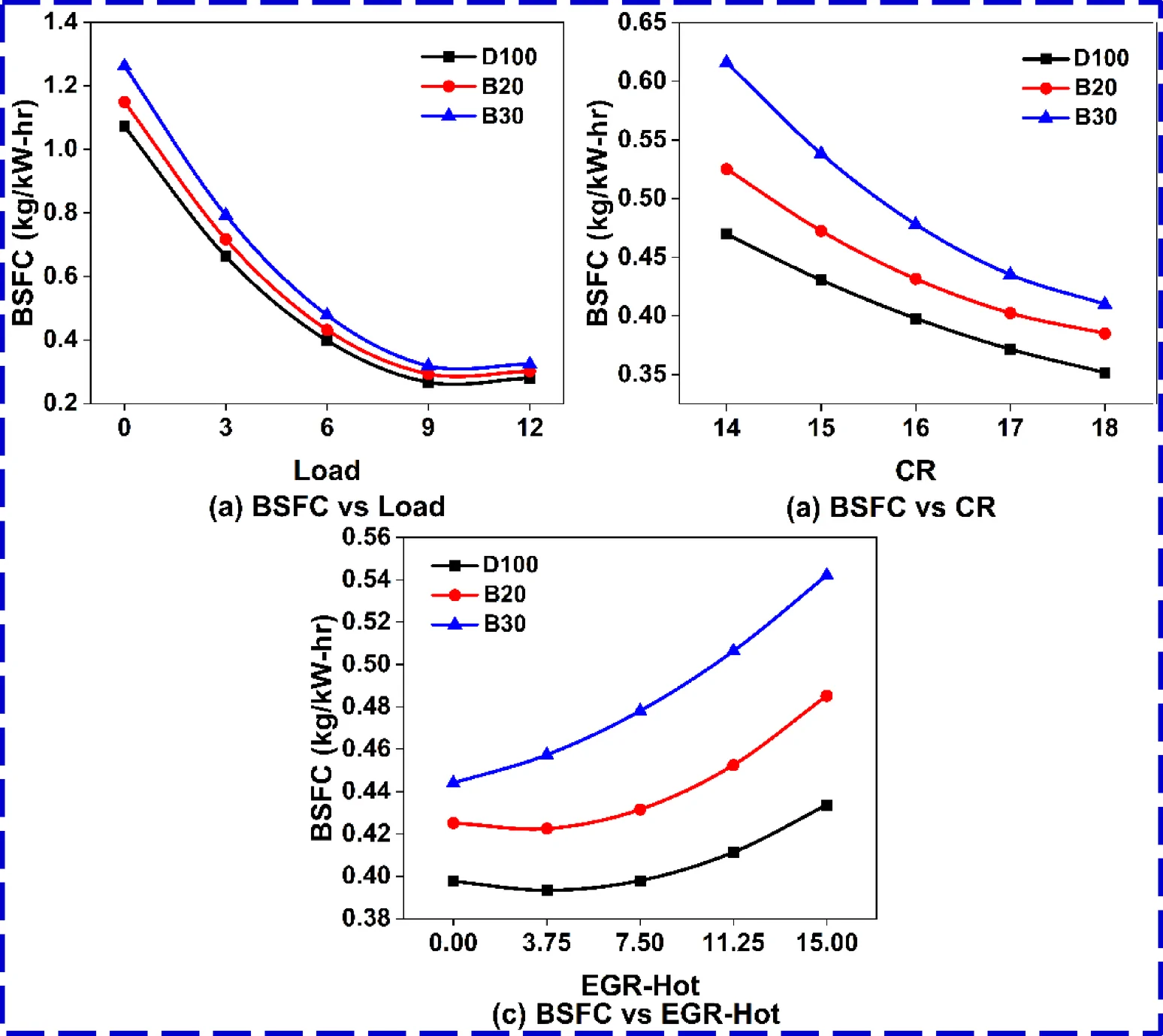
Significance of BSFC (**a**) BSFC vs. Load, (**b**) BSFC vs. CR, (**c**) BSFC vs. EGR-Hot.

Figure 6 (a) demonstrates the variation of BSFC with load for different fuel blends. At zero load, the BSFC was 1.071 kg/kW-hr for D100, 1.149 for B20, and 1.262 for B30. At full load (12 kg), it decreased to 0.279, 0.301, and 0.324 kg/kW-hr for diesel, B20, and B30, respectively. The BSFC for B30 increased by 18.52% and 7.95% compared to Diesel and B20, respectively. Figure 6 (b) demonstrates the variation in BSFC with change in CR for different fuel blends. At lower CR value i.e. CR 14, the BSFC was found to be 0.469 kg/kW-hr for Diesel, 0.525 kg/kW-hr for B20, & 0.616 kg/kW-hr for B30. At higher CR value i.e. CR 18, the BSFC decreased to 0.351 kg/kW-hr, 0.385 kg/kW-hr, and 0.410 kg/kW-hr, respectively, for Diesel, B20, & B30. The BSFC for B30 increased by 22.56% and 9.66% compared to Diesel and B20, respectively. Figure 6 (c) demonstrates the variation of BSFC with EGR-Hot for all fuels. At 0% EGR-Hot, the BSFC was found to be 0.397 kg/kW-hr for Diesel, 0.425 kg/kW-hr for B20, and 0.444 kg/kW-hr for B30. At 15% EGR-Hot, the BSFC increased to 0.433 kg/kW-hr, 0.485 kg/kW-hr, and 0.542 kg/kW-hr, respectively, for Diesel, B20, & B30. The BSFC for the B30 increased by 19.35% and 8.99% compared to Diesel and B20, respectively. The BSFC for higher biodiesel blends was always more than diesel due to their lesser calorific value, more density, and viscosity. These properties lead to poorer atomization & reduced combustion efficiency, requiring extra fuel to generate same power output. The oxygen content improves combustion but cannot fully offset the lower energy content^37,38^. The variation in BSFC between diesel and Karanja biodiesel can be explained by differences in their physical & combustion properties. Biodiesel has greater viscosity and reduced volatility than diesel, resulting in larger fuel droplets & poorer atomization. As a result, combustion efficiency decreases, especially when the available time to complete each engine cycle is limited. The effects are more noticeable under elevated engine speeds & loads. Under such conditions, mixing-controlled combustion dominates over premixed combustion. The shorter interaction time between the fuel spray and in-cylinder air, combined with slower chemical reaction rates, further affects the combustion process of biodiesel blends compared to diesel^39,40^.

#### Impact of operating parameters on BTHE

**Fig. 7:**
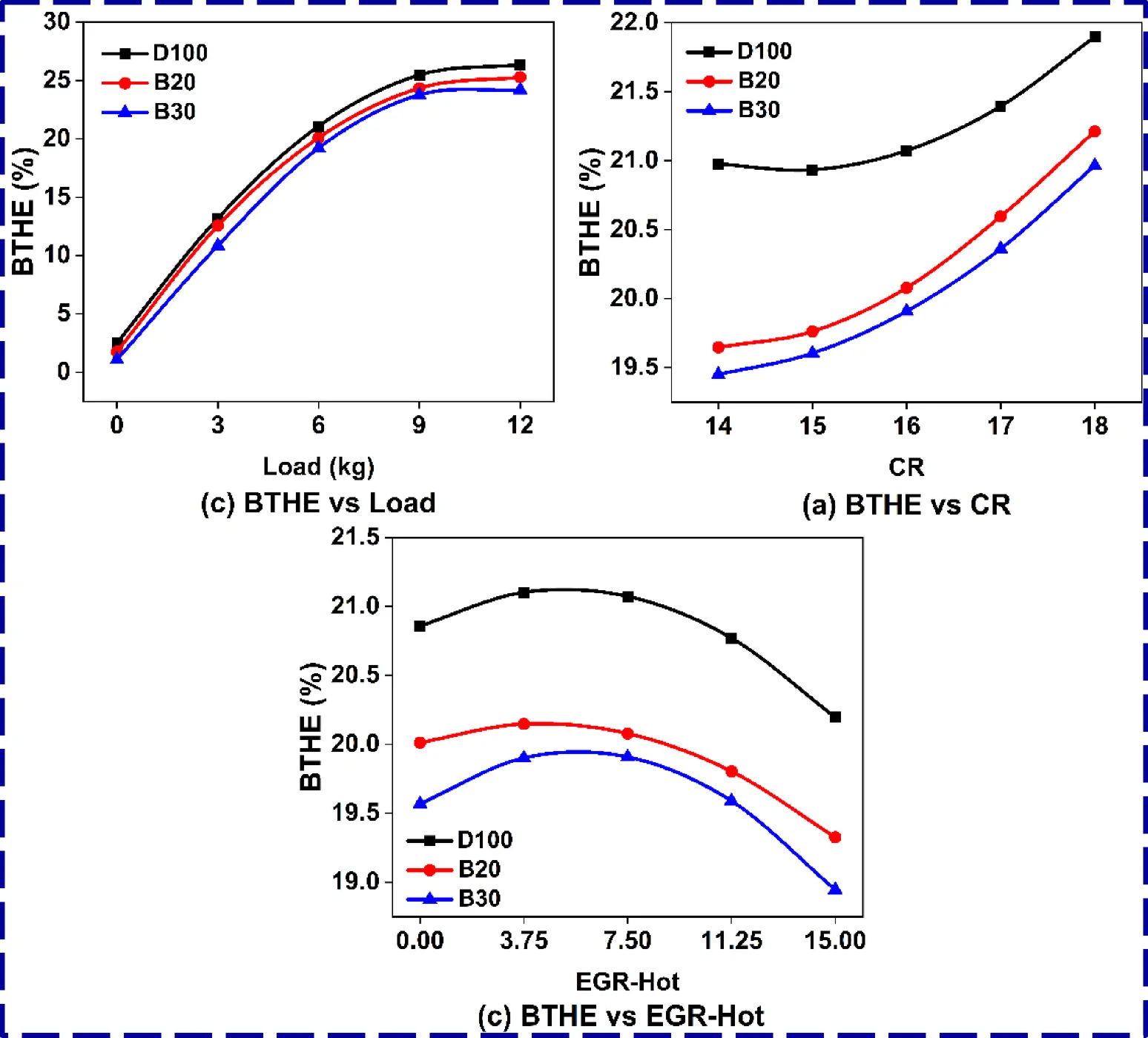
Significance of BHTE (**a**) BTHE vs. Load, (**b**) BTHE vs. CR, (**c**) BTHE vs. EGR-Hot.

Figure 7 (a) reveals the difference of BTHE with Load for different fuel blends. At 0 kg load, the BTHE was found to be 2.493% for Diesel, 1.751% for B20, and 1.067% for B30. At full Load 12 kg, the BTHE increased to 26.349, 25.285, and 24.185%, respectively, for D100, B20, and B30. The BTHE for the B30 decreased by 10.74% & 5.16% relative to Diesel and B20, respectively. Figure 7 (b) demonstrates how BTHE changes with CR for various fuel blends. At lower CR value i.e. CR 14, the BTHE was found to be 20.975% for Diesel, 19.645% for B20, and 19.449% for B30. At higher CR value i.e. CR 18, the BTHE increased to 21.897%, 0 21.211%, and 20.963%, respectively, for Diesel, B20, and B30. The BTHE for the B30 decreased by 5.63 and 4.68% compared to D100 & B20, respectively. Figure 7 (c) demonstrates the variation of BTHE with EGR-Hot for all fuels. At 0% EGR-Hot, the BTHE was found to be 20.857% for Diesel, 20.011% for B20, and 19.567% for B30. At 15% EGR-Hot, the BTHE decreased to 20.195, 19.325, and 18.945%, respectively, for D100, B20, and B30. The BTHE for the B30 decreased by 5.85 and 4.45% compared to diesel and B20, respectively. This behavior may be explained by intrinsic characteristics of biodiesel, including its comparatively lower energy content, higher viscosity and density, increased volatility, shorter ignition delay, and relatively poorer spray atomization^41^.

Biodiesel blends exhibited lower BTHE compared to diesel, primarily due to their higher viscosity and the additional energy required for fuel vaporization. Furthermore, elevated density & viscosity of biodiesel adversely affects atomization process, promoting formation of larger fuel droplets and leading to a less uniform fuel air mixture^42,43^. The inclusion of oxygen within fuel molecules enhances combustion efficiency by promoting more complete oxidation. At higher engine loads, the increased in-cylinder air temperature improves fuel evaporation and air–fuel mixing. Consequently, BTHE of higher biodiesel blends tends to improve under high-load conditions^38^.

#### Impact of operating parameters on CPMax

**Fig. 8:**
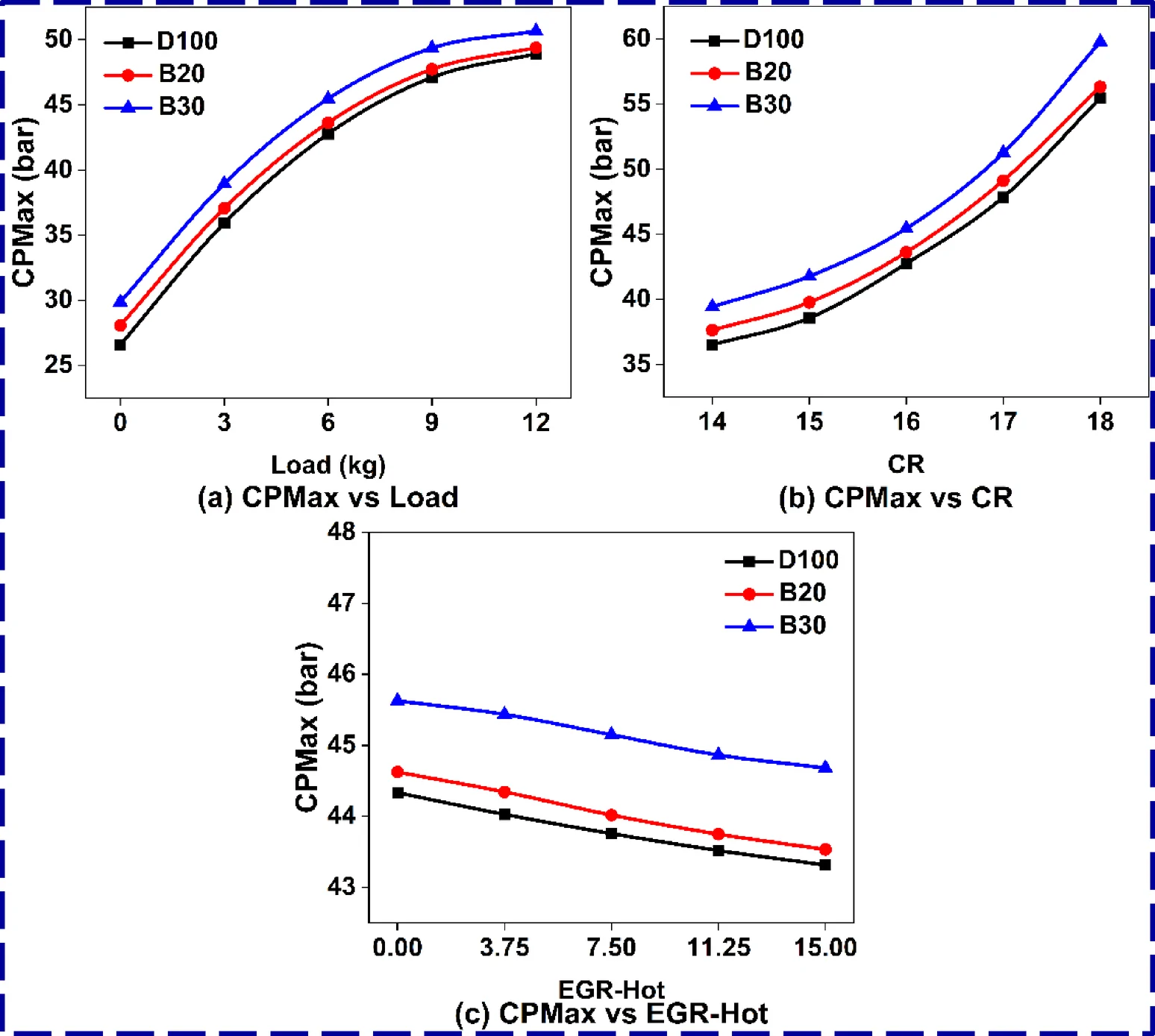
Significance of CPMax (**a**) CPMax vs. Load, (**b**) CPMax vs. CR, (**c**) CPMax vs. EGR-Hot.

Figure 8 (a) demonstrates difference of CPMax with load for diverse fuel blends. At 0 kg load, CPMax was found to be 26.560 bar for D100, 28.052 bar for B20, and 29.840 bar for B30. At full Load 12 kg, the CPMax increased to 48.904 bar, 49.372 bar, and 50.644 bar, respectively, for Diesel, B20, and B30. The CPMax for the B30 increased by 3.43% and 2.51% compared to Diesel and B20, respectively.

Figure 8 (b) depicts the trend of CPMax with respect to CR for various fuel blends. At lower CR value i.e. CR 14, the CPMax was found to be 36.514 bar for diesel, 37.628 bar for B20, and 39.430 bar for B30. At higher CR value i.e. CR 18, the CPMax increased to 55.470 bar, 56.326 bar, and 59.774 bar, respectively, for D100, B20, and B30. The CPMax for the B30 increased by 6.94% and 4.7% compared to Diesel and B20, respectively. Figure 8 (c) demonstrates variation of CPMax with EGR-Hot for dissimilar fuel blends. At no EGR-Hot, the CPMax was found to be 44.330 bar for Diesel, 44.622 bar for B20, and 45.624 bar for B30. At 15% EGR-Hot, the CPMax decreased to 43.314 bar, 43.532 bar, and 44.68 bar, respectively, for Diesel, B20, and B30. The CPMax for the B30 increased by 3.01% and 2.43% compared to Diesel and B20, respectively. With an increase in engine load, CPMax shows a steady rise across all fuel blends. This occurs because higher loads require greater fuel injection, resulting in increased in-cylinder pressure. The chemically bound oxygen in biodiesel droplets facilitates improved oxidation during combustion, thereby increasing in-cylinder temperature and pressure levels^43–45^. The higher cetane number of biodiesels leads to an earlier rise in cylinder pressure. This indicates improved combustion behavior, particularly under conditions of elevated pressure and temperature during fuel injection and at the start of combustion. At elevated engine loads, residual gases & cylinder wall temperatures increase, resulting in a higher in-cylinder charge temperature during injection. This enhances evaporation and air–fuel mixing, especially for fuels with lower volatility^46,47^.

#### Impact of operating parameters on NHR

**Fig. 9:**
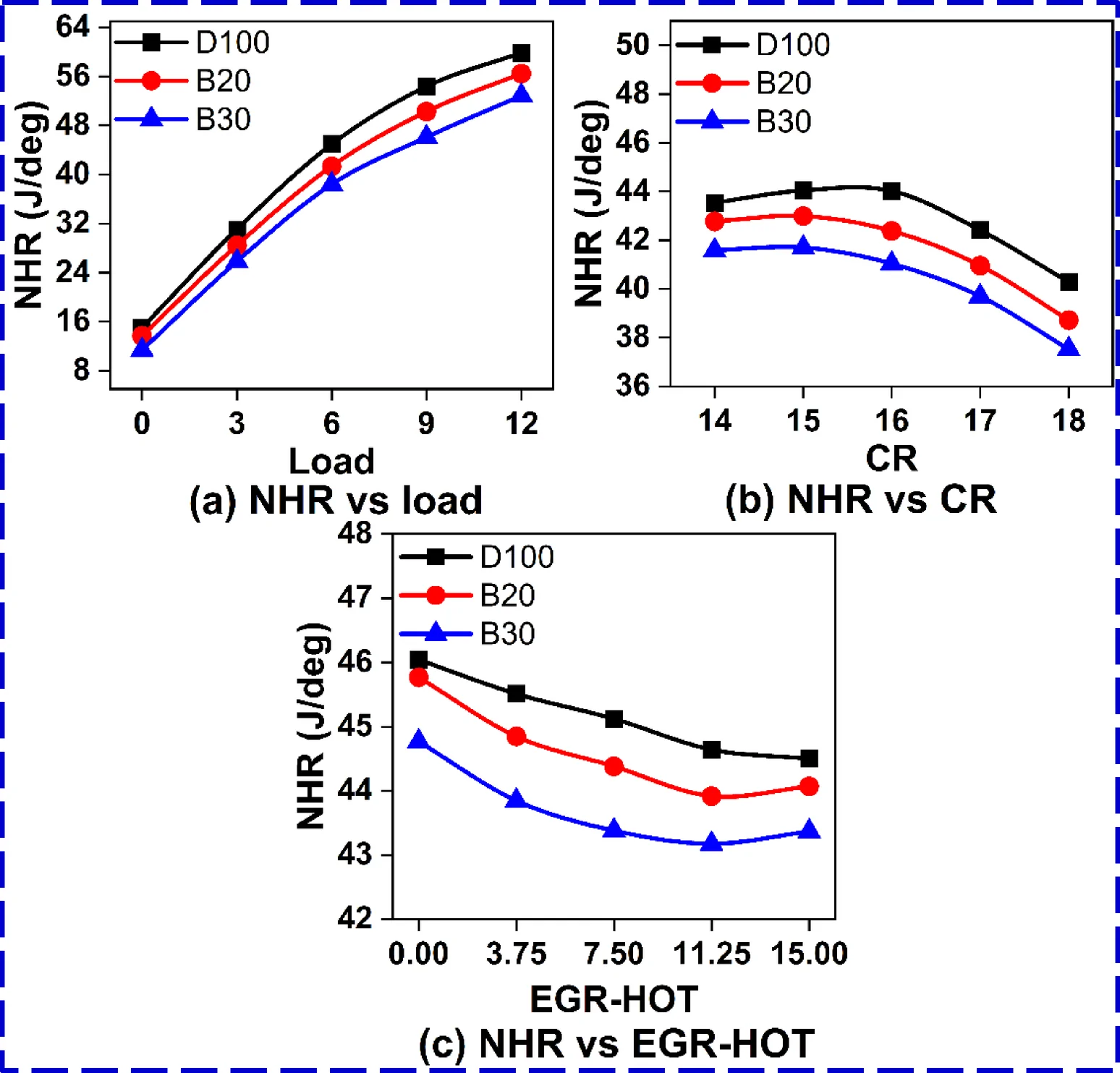
Significance of NHR (**a**) NHR vs. Load, (**b**) NHR vs. CR, (**c**) NHR vs. EGR-Hot.

Figure 9 (a) demonstrates difference of NHR with load for diverse fuel blends. At 0 kg load, NHR was found to be 14.97 J/deg for D100, 13.67 J/deg for B20, and 11.35 J/deg for B30. At full Load 12 kg, the NHR increased to 59.84 J/deg, 56.46 J/deg, and 52.85 J/deg, respectively, for Diesel, B20, and B30. The NHR for the B20 and B30 decreased by 7.31% and 14.94% compared to Diesel. Figure 9 (b) depicts the trend of NHR with respect to CR for various fuel blends. At lower CR value i.e. CR 14, the NHR was found to be 43.53 J/deg for diesel, 42.77 J/deg for B20, and 41.57 J/deg for B30. At higher CR value i.e. CR 18, the NHR decreased to 40.28 J/deg, 38.72 J/deg, and 37.52 J/deg, respectively, for D100, B20, and B30. The NHR for the B20 and B30 decreased by 3.02% and 5.95% compared to Diesel. Figure 9 (c) demonstrates variation of NHR with EGR-Hot for dissimilar fuel blends. At no EGR-Hot, the NHR was found to be 46.04 J/deg for Diesel, 45.76 J/deg for B20, and 44.76 J/deg for B30. At 15% EGR-Hot, the NHR decreased to 44.49 J/deg, 44.06 J/deg, and 43.36 J/deg, respectively, for Diesel, B20, and B30. The NHR for the B20 and B30 decreased by 1.25% and 3.22% compared to Diesel. All biodiesel blends exhibited a reduction in NHR compared to conventional diesel across all engine operating conditions. This behavior can be attributed to the shorter ignition delay and reduced premixed combustion phase associated with biodiesel fuels. In contrast, diesel operation involves a longer ignition delay, which allows greater fuel accumulation prior to combustion, resulting in a higher heat release rate (HRR). Biodiesel and its blends initiate combustion earlier due to their shorter ignition delay, leading to an earlier onset of heat release. However, the overall heat release rate is lower than that of diesel. This is primarily due to the relatively slower combustion process and the inherent oxygen content in biodiesel, which promotes more controlled and gradual energy release^48,49^. This behavior is also primarily attributed to the lower calorific value of biodiesel, which results in a reduced heat release rate during combustion. Additionally, the higher cetane number of the B30 biodiesel blend shortens the ignition delay, leading to an earlier start of combustion^37,50^.

Combustion parameters such as ignition delay, rate of heat release, and in-cylinder pressure rise significantly influence engine vibration. A longer ignition delay leads to rapid premixed combustion, resulting in a sharp pressure rise and higher vibration amplitudes. Similarly, higher heat release rates and steep pressure gradients intensify structural excitation of engine components. In contrast, shorter ignition delay and controlled combustion phasing promote smoother pressure development, thereby reducing vibration levels. Thus, vibration signals serve as an indirect indicator of combustion quality and stability in the engine.

### Impact of operating parameters on emission

#### Impact of operating parameters on NOx

**Fig. 10:**
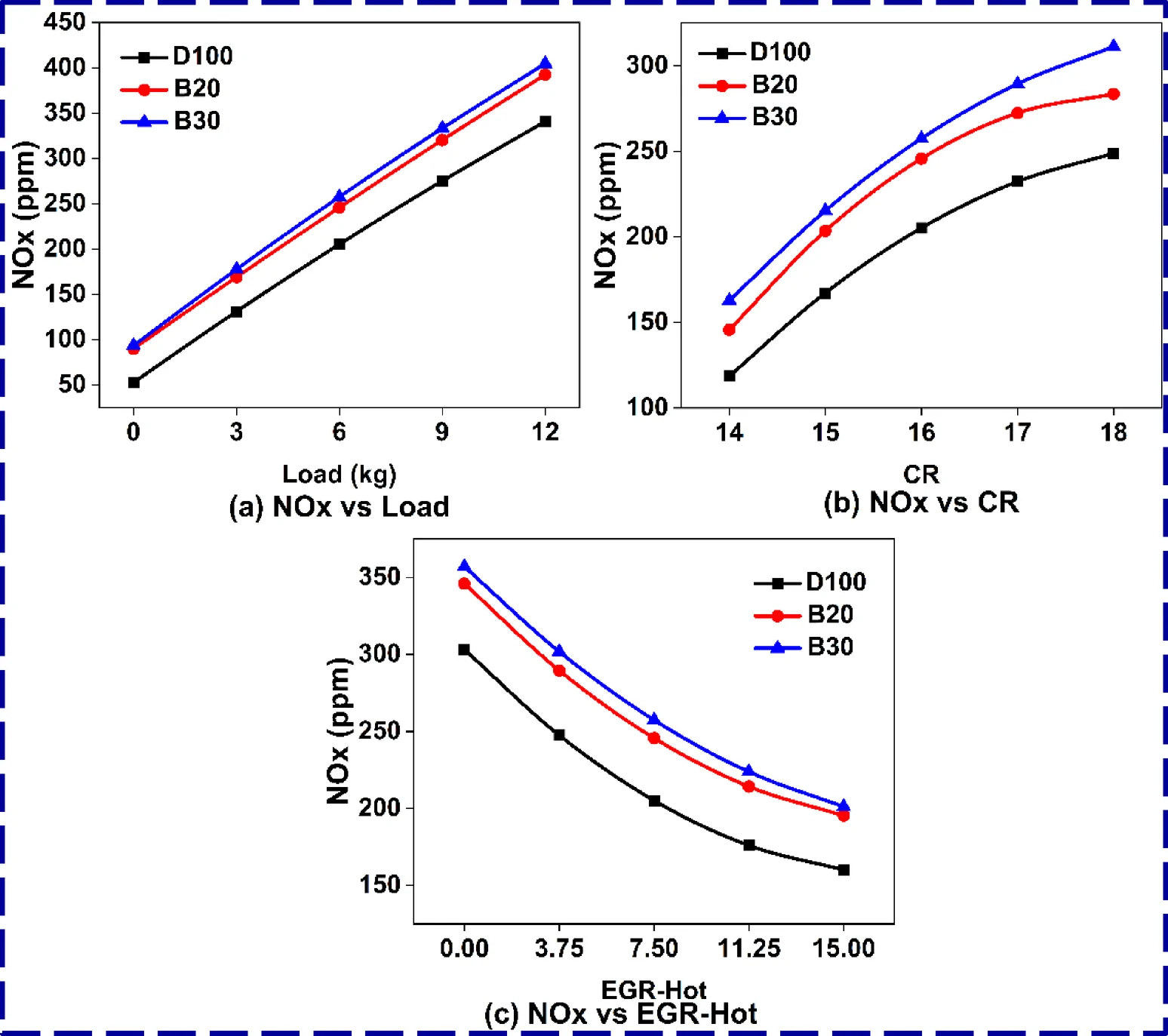
Significance of NOx (**a**) NOx vs. Load, (**b**) NOx vs. CR, (**c**) NOx vs. EGR-Hot.

Figure 10 (a) displays changes in Nitrous Oxides (NOx) as load varies for all fuel blends. At 0 kg load, the NOx was found to be 52.727 ppm for D100, 89.872 ppm for B20, and 93.454 ppm for B30. At full Load 12 kg, the NOx increased to 340.527 ppm, 392.272 ppm, and 404.454 ppm, respectively, for Diesel, B20, and B30. The NOx for the B30 increased by 15.8% and 3.01% compared to Diesel and B20, respectively. Figure 10 (b) demonstrates changes in NOx with CR for diverse fuel blends. At lower CR value i.e. CR 14, the NOx was found to be 118.527 ppm for Diesel, 145.672 ppm for B20, and 162.854 ppm for B30. At higher CR value i.e. CR 18, the NOx increased to 248.727 ppm, 283.472 ppm, and 311.054 ppm, respectively, for D100, B20, and B30. The NOx for the B30 increased by 20.03% and 8.86% compared to Diesel and B20, respectively. Figure 10 (c) demonstrates the variation of NOx with EGR-Hot for all fuel blends. At no EGR-Hot, the NOx was found to be 303.127 ppm for D100, 345.872 ppm for B20, and 356.854 ppm for B30. At 15% EGR-Hot, NOx decreased to 160.127 ppm, 195.272 ppm, and 201.054 ppm, respectively, for Diesel, B20, and B30. The NOx for the B30 increased by 20.3% and 2.87% compared to Diesel and B20, respectively. The NOx emissions augmented with engine load for tested fuels because of additional exhaust gas temperatures & longer residence times at elevated loads. However, biodiesel & its blends produced lesser NOx emissions than diesel at more loads^51,52^. The NOx emission for biodiesel blends increased mainly due to more oxygen availability & greater cetane number of fuel. These factors promote more efficient combustion within the chamber, leading to higher cylinder combustion intensity and an increase in the adiabatic flame temperature^53^. Research findings on NOx emissions from biodiesel-fueled engines are mixed. The O_2_ content in biodiesel is often considered a factor contributing to increased NOx formation, as it can promote more complete combustion and higher local temperatures. However, some analyses suggest that the overall oxygen-to-fuel mass ratio in biodiesel blends may not necessarily exceed that of conventional diesel, indicating that fuel-bound oxygen alone may not fully explain the rise in NOx emissions. In certain cases, moderate biodiesel blends show higher NOx emissions at rated load, while increasing the biodiesel proportion further may reduce brake-specific NOx emissions. Additionally, NOx emissions tend to increase with higher biodiesel concentration across various load conditions. At lower engine loads, poorer air–fuel mixing & reduced in-cylinder temperatures can lead to lesser NO_x_ emissions compared to higher engine loads. Increased NOx formation has also been associated with enhanced prompt NOx mechanisms during biodiesel combustion^54–56^.

#### Impact of operating parameters on HC

**Fig. 11:**
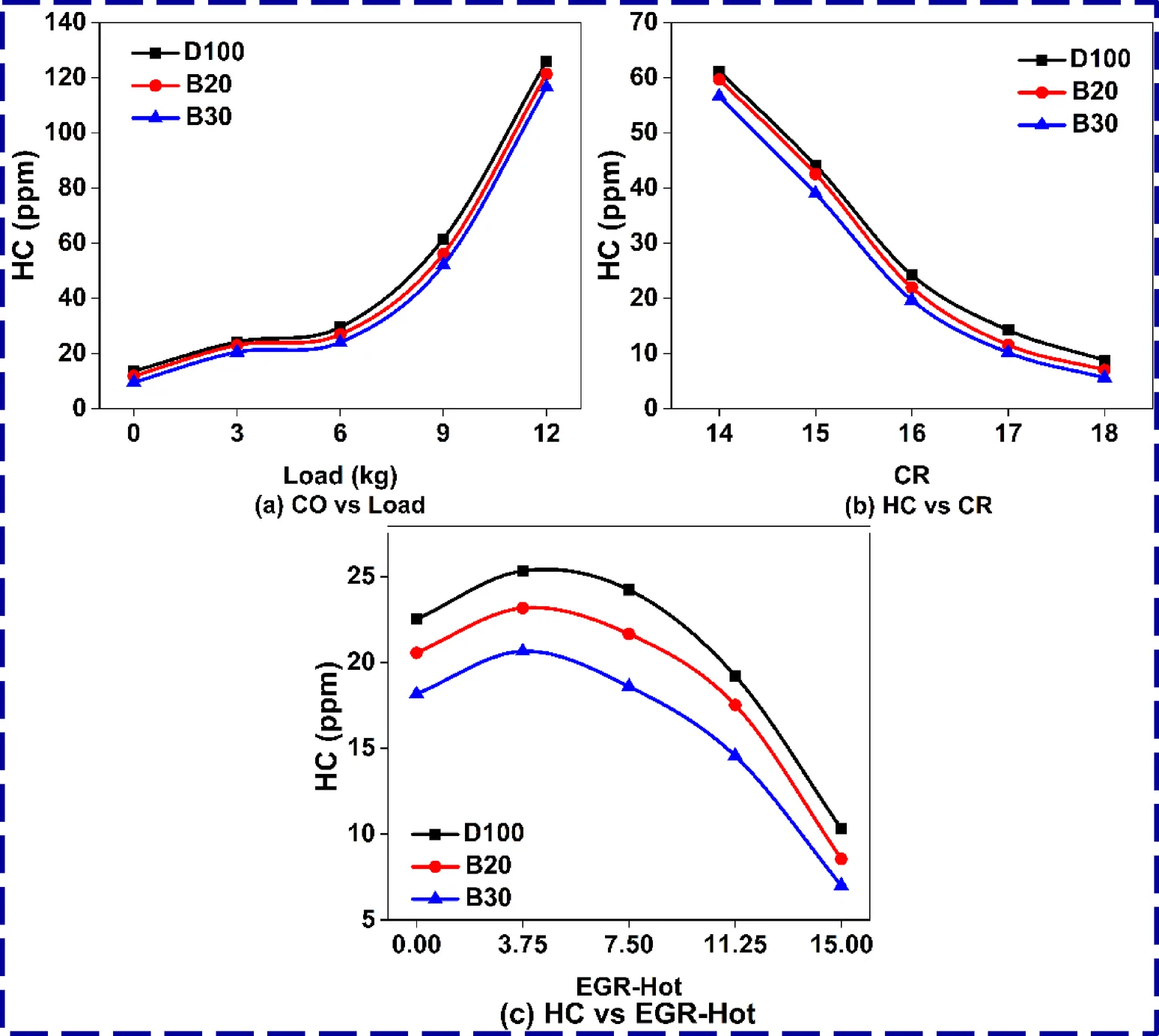
Significance of HC (**a**) HC vs. Load, (**b**) HC vs. CR, (**c**) HC vs. EGR-Hot.

Figure 11 (a) reveals variation of HC with Load for all fuel blends. At 0 kg load, the HC were found to be 13.572 ppm for D100, 11.831 ppm for B20, and 9.564 ppm for B30. At full Load 12 kg, the HC increased to 125.809 ppm, 121.354 ppm, and 118.514 ppm, respectively, for Diesel, B20, and B30. The HC for the B30 decreased by 13.3% and 6.43% compared to Diesel and B20, respectively. Figure 11 (b) demonstrates the variation of HC with CR for different fuel blends. At lower CR value i.e. CR 14, the HC was found to be 61.109 ppm for D100, 59.652 ppm for B20, and 56.615 ppm for B30. At higher CR value i.e. CR 18, the HC decreased to 8.783 ppm, 7.014 ppm, and 6.765 ppm, respectively, for Diesel, B20, and B30. The NOx for the B30 decreased by 15.35% and 7.99% compared to Diesel and B20, respectively.

Figure 11 (c) demonstrates the variation of HC with EGR-Hot for all fuel blends. At no EGR-Hot, the HC was found to be 22.509 ppm for D100, 20.568 ppm for B20, and 18.165 ppm for B30. At 15% EGR-Hot, the HC decreased to 10.309 ppm, 8.561 ppm, and 7.005 ppm, respectively, for Diesel, B20, and B30. The HC for the B30 diminished by 28.62% & 15.8% compared to D100 and B20, respectively. Lower HC emissions are observed at partial loads, whereas higher engine loads result in increased HC formation because elevated fuel injection reduces the oxygen-to-fuel ratio during combustion. Diesel shows higher HC emissions than biodiesel blends, as incomplete combustion is the main source. The greatest HC reduction was observed for neat B30 biodiesel^57–59^.

#### Impact of operating parameters on CO

**Fig. 12:**
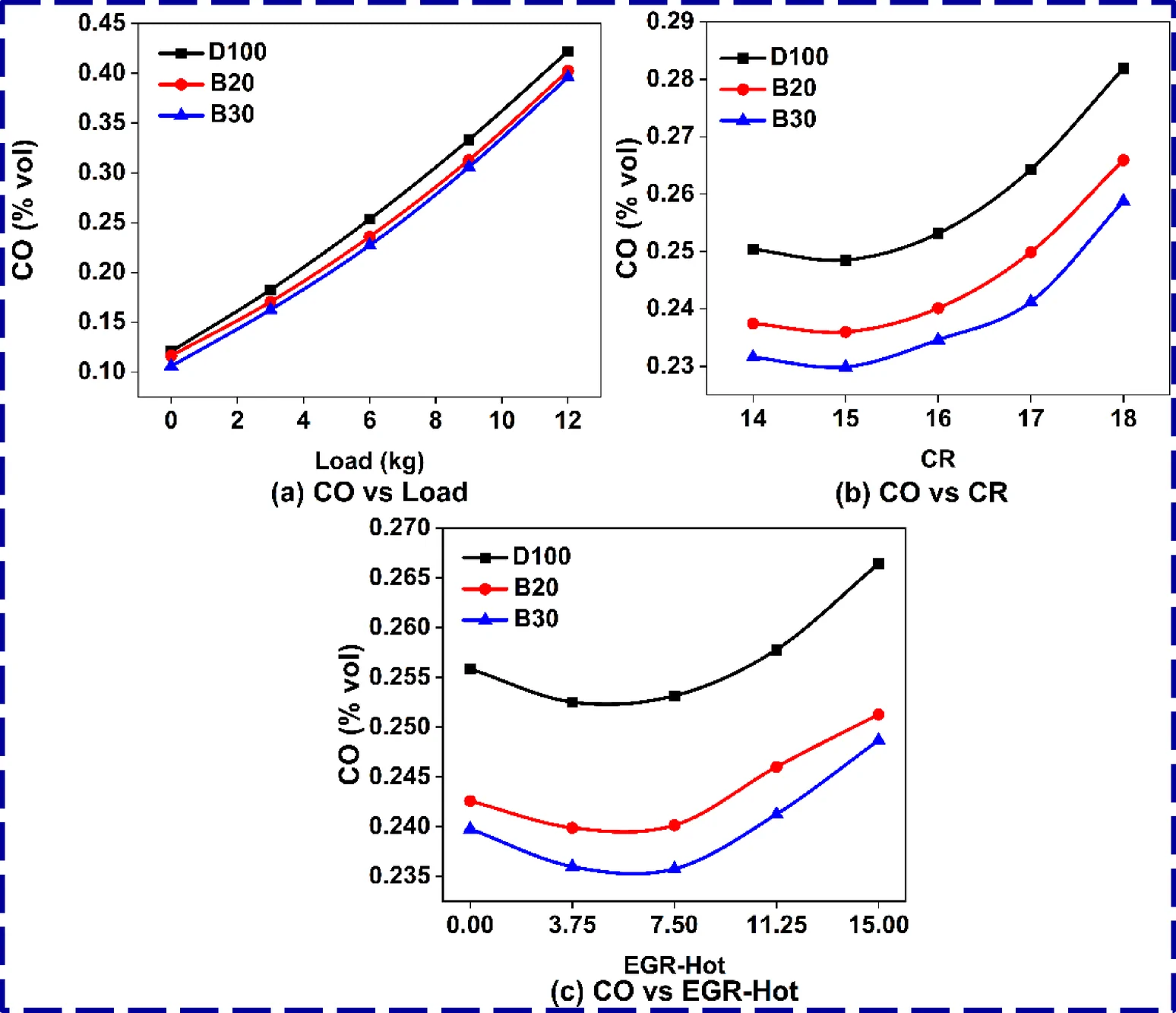
Significance of CO (**a**) CO vs. Load, (**b**) CO vs. CR, (**c**): CO vs. EGR-Hot.

Figure 12 (a) demonstrates the variation of CO with load for all fuel blends. At zero load, the CO emissions were measured as 0.1204%vol for diesel, 0.116%vol for B20, and 0.112%vol for B30. When the load was increased to 12 kg, CO emissions rose to 0.421%vol for diesel, 0.402%vol for B20, and 0.395%vol for B30. Compared to diesel and B20, the B30 blend exhibited a reduction in CO emissions of 6.58% and 1.68%, respectively. Figure 12 (b) demonstrates the variation of CO with CR for diverse fuel blends. At a lower (CR = 14), the CO emissions were recorded as 0.250%vol for diesel, 0.237%vol for B20, and 0.231%vol for B30. When the CR was increased to 18, CO emissions rose to 0.281%vol for diesel, 0.265%vol for B20, and 0.258%vol for B30. Overall, the B30 blend exhibited a reduction in CO emissions of 8.94% and 2.76% compared to diesel and B20, respectively.

Figure 12 (c) demonstrates the variation of CO with EGR-Hot for all fuel blends. Under 0% hot EGR conditions, CO emissions were measured as 0.255%vol for diesel, 0.242%vol for B20, and 0.239%vol for B30. When the hot EGR rate was increased to 15%, CO emissions rose to 0.266%vol for diesel, 0.251%vol for B20, and 0.248%vol for B30. The CO for the B30 decreased by 7.13% and 1.04% compared to Diesel and B20, respectively. CO emissions increased with engine load for all tested fuels. However, B30 blend exhibited 10.32% drop in CO emissions compared to diesel, which can be ascribed to its more O_2_ content and improved cetane number, promoting more complete combustion^60^. The more cetane number shortens ignition delay, while the oxygen in biodiesel enhances combustion, raising in-cylinder temperature and promoting CO-to-CO_2_ conversion^61^. This also may be occurred because higher loads create a richer air–fuel mixture, leading to incomplete combustion. However, blended biodiesel fuels, especially B30, display lesser CO emissions because of their higher O_2_ content, which encourages additional complete combustion & converts CO to CO_2_^62,63^.

#### Impact of operating parameters on smoke opacity

**Fig. 13:**
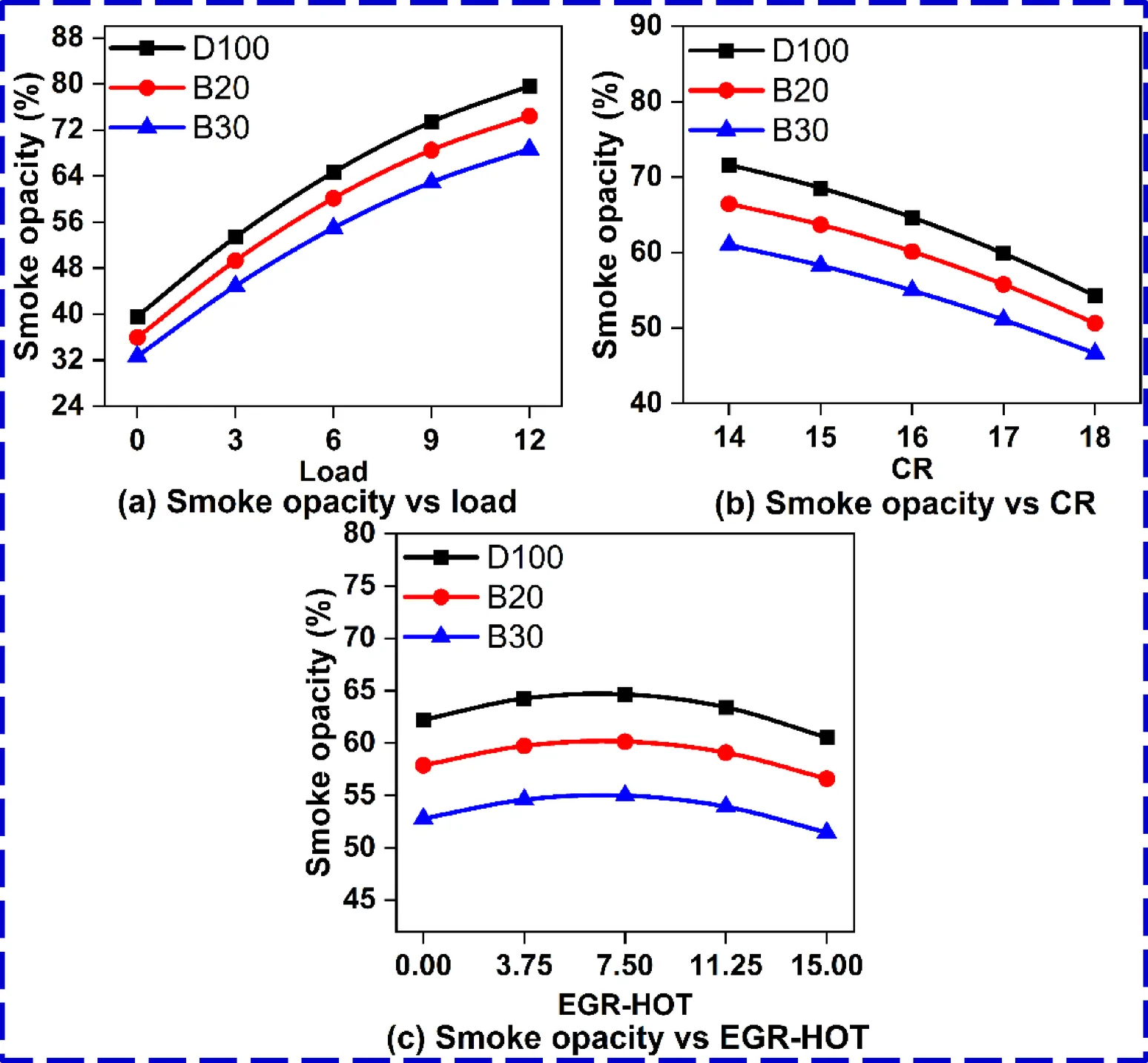
Significance of smoke opacity (**a**) smoke opacity vs. Load, (**b**) smoke opacity vs. CR, (**c**): smoke opacity vs. EGR-Hot.

Figure 13 (a) demonstrates the variation of smoke opacity with load for all fuel blends. At zero load, the smoke opacity emissions were measured as 39.61%vol for diesel, 35.96%vol for B20, and 32.68%vol for B30. When the load was increased to 12 kg, smoke opacity emissions rose to 79.62%vol for diesel, 74.41%vol for B20, and 68.65%vol for B30. Compared to diesel and B20, the B30 blend exhibited a reduction in smoke opacity emissions of 14.98% and 8.39%, respectively. Figure 13 (b) demonstrates the variation of smoke opacity with CR for diverse fuel blends. At a lower (CR = 14), the smoke opacity emissions were recorded as 71.64%vol for diesel, 66.44%vol for B20, and 60.99%vol for B30. When the CR was increased to 18, smoke opacity emissions decreased to 54.29%vol for diesel, 50.64%vol for B20, and 46.64%vol for B30. Overall, the B30 blend exhibited a reduction in smoke opacity emissions of 14.75% and 8.32% compared to diesel and B20, respectively. Figure 13 (c) demonstrates the variation of smoke opacity with EGR-Hot for all fuel blends. Under 0% hot EGR conditions, smoke opacity emissions were measured as 62.21%vol for diesel, 57.88%vol for B20, and 52.78%vol for B30. When the hot EGR rate was increased to 15%, smoke opacity emissions decreased to 60.23%vol for diesel, 56.59%vol for B20, and 51.45%vol for B30. The B30 blend exhibited a reduction in smoke opacity emissions of 15.01% and 8.75% compared to diesel and B20, respectively. It was observed that all biodiesel blends produced lower smoke emissions than diesel fuel under all operating conditions. This reduction is mainly attributed to the higher oxygen content in biodiesel, which promotes more complete combustion and consequently minimizes smoke formation^64,65^.

## Conclusion

This study experimentally evaluated the performance, combustion, emissions, and vibration characteristics of a VCR diesel engine fueled with diesel and Karanja biodiesel blends (B20 and B30) under varying load, CR, and EGR-HOT conditions. The integrated approach provided a comprehensive understanding of engine behavior by correlating vibration characteristics with performance and emission parameters.

- Biodiesel blends (especially B30) reduced engine vibration and improved emission characteristics compared to diesel across all operating conditions.
- Vibration behavior is strongly influenced by operating parameters, increasing with load, decreasing with CR, and showing a nonlinear trend with EGR-HOT.
- Combustion characteristics improved with higher peak pressure and controlled heat release, though performance slightly declined due to lower calorific value.
- Engine vibration analysis revealed that vibration increases with load and decreases with higher compression ratios, while under EGR-HOT conditions it decreases initially and then increases at higher rates. Compared to diesel, RMS acceleration decreased by 4.3% and 8.49% under load variation, 3.01% and 7.18% under CR variation, and 2.66% and 6.75% under EGR-HOT conditions for B20 and B30, respectively.
- Emission results showed that the B30 blend reduced HC, CO, and smoke emissions by 13.3%, 6.58%, and 17.98%, respectively, compared to diesel, although NOx emissions increased by 15.8%, primarily due to the higher oxygen content and improved combustion, which enhance oxidation but promote higher in-cylinder temperatures leading to increased NOx formation.
- Combustion analysis indicated a 3.43% increase in CPmax and a 14.94% decrease in NHR, primarily due to the shorter ignition delay and oxygenated nature of biodiesel, which promotes earlier and more controlled combustion.
- Performance results showed a 10.74% reduction in BTHE and an 18.52% increase in BSFC compared to diesel, primarily due to the lower calorific value and higher viscosity of biodiesel, which affect fuel atomization and combustion efficiency.

Overall, the use of biodiesel blends improves emission and vibration characteristics due to enhanced combustion, but slightly compromises engine performance because of their lower calorific value and fuel properties.

### Future scope

Future work can focus on improving efficiency and emission performance through hydrogen induction and the use of suitable fuel additives. Further investigation into a greater variety of biodiesel blends and alternative fuels will enable condition monitoring of diesel engines using engine block vibrations in the future. Vibration data could be subjected to sophisticated machine learning algorithms for more precise defect diagnosis and predictive maintenance. To improve the generalizability of the results, additional research on multi-cylinder engines and in other operating environments could be done. Continuous and remote engine diagnostics could be made possible by integrating Internet of Things (IoT) technologies with real-time monitoring systems. The long-term consequences of biodiesel blends on engine wear and durability, as well as their implications on the environment, could be subjected the future research. Ultimately, more sustainable engine technologies can be achieved by reducing emissions and increasing fuel efficiency through the use of sophisticated management systems to optimize engine characteristics.

## Data Availability

The datasets generated during and/or analyzed during the current study are available from the corresponding author on reasonable request.
